# Cathodic generation of reactive (phenylthio)difluoromethyl species and its reactions: mechanistic aspects and synthetic applications

**DOI:** 10.3762/bjoc.18.88

**Published:** 2022-07-20

**Authors:** Sadanobu Iwase, Shinsuke Inagi, Toshio Fuchigami

**Affiliations:** 1 Department of Electronic Chemistry, Tokyo Institute of Technology, Yokohama 226-8502, Japan; 2 Department of Chemical Science and Engineering, Tokyo Institute of Technology, Yokohama 226-8502, Japan

**Keywords:** bis(phenylthio)difluoromethane, cathodic reduction, deuteration, *o*-phthalonitrile mediator, (phenylthio)difluoromethylation, (phenylthio)difluoromethyl radical

## Abstract

The cathodic reduction of bromodifluoromethyl phenyl sulfide (**1**) using *o*-phthalonitrile as a mediator generated the (phenylthio)difluoromethyl radical, which reacted with α-methylstyrene and 1,1-diphenylethylene to provide the corresponding adducts in moderate and high yields, respectively. In contrast, chemical reduction of **1** with SmI_2_ resulted in much lower product yields. The detailed reaction mechanism was clarified based on the cathodic reduction of **1** in the presence of deuterated acetonitrile, CD_3_CN.

## Introduction

**Scheme 1:**
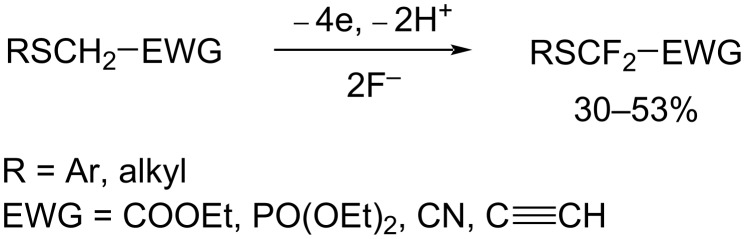
Electrochemical *gem*-difluorination of sulfides bearing α-electron-withdrawing groups.

**Scheme 2:**
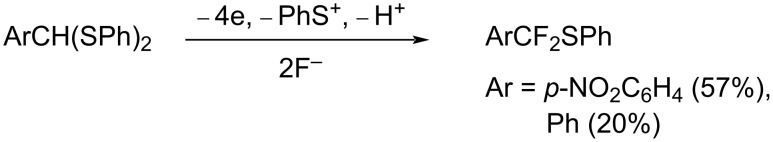
Electrochemical gem-difluorodesulfurization of dithioacetals.

**Scheme 3:**
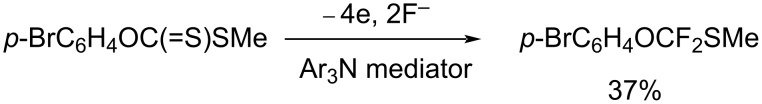
Electrochemical *gem*-difluorodesulfurization of dithiocarbonate.

Organofluorine compounds containing a difluoromethylene group have been of much interest from biological aspects since the difluoromethylene group is isopolar and isosteric with an ether oxygen [1–2]. Particularly, organic molecules bearing a (arylthio)difluoromethyl group (ArSCF_2_) have potential biological applications such as anti-HIV-1 reverse transcriptase inhibitors and agrochemical applications [3–4]. Reurakul and Pohmakotr et al. carried out the reaction of PhSCF_2_Br with SmI_2_ in THF/iPrOH to generate PhSCF_2_ radicals followed by trapping with various olefins in moderate yields [5]. Prakash et al. also achieved fluoride-induced nucleophilic (phenylthio)difluoromethylation of carbonyl compounds using PhSCF_2_SiMe_3_ [6]. Quite recently, Shen et al., developed various nucleophilic, electrophilic, and radical difluoromethylthiolating reagents [1]. However, these methods require various metal and organometallic reagents. On the other hand, electrochemical organic synthesis is a metal-free process and does not require any hazardous reagents and it produces less waste than conventional chemical syntheses. Therefore, electrochemical synthesis is desirable from an aspect of green chemistry [7–10]. In this context, we have developed various electrochemical methodologies for efficient selective fluorination [11–12] and molecular conversion of organofluorine compounds to date [13–18]. We have also achieved the *gem*-difluorination of sulfides bearing various electron-withdrawing groups at the α-position (Scheme 1) [19–21]. Furthermore, we also succeeded in the electrochemical *gem*-difluorodesulfurization of dithioacetals and dithiocarbonate (Scheme 2 and Scheme 3) [22–23].

In this work, we have studied the electrochemical generation of (phenylthio)difluoromethyl reactive species from bromodifluoromethyl phenyl sulfide and their synthetic application as well as mechanistic aspects.

## Results and Discussion

### Cathodic reduction of bromodifluoromethyl phenyl sulfide (**1**)

At first, the reduction potential (*E*_p_^red^) of bromodifluoromethyl phenyl sulfide (**1**) was measured by cyclic voltammetry in an anhydrous acetonitrile (MeCN) solution containing Bu_4_NClO_4_ (0.1 M) using a platinum electrode. One irreversible reduction peak was observed at −2.4 V vs SSCE at a scan rate of 100 mV/s. Even at a much higher scan rate of 500 mV/s, the reduction peak was irreversible. Since the reduction potentials (*E*_p_^red^) of CF_3_Br and PhCF_2_Cl are −1.55 V (Pt cathode) and −2.11 V vs SCE (hanging Hg drop cathode), respectively [24–25], the reduction potential of **1** was found to be similar to that of PhCF_2_Cl.

**Scheme 4:**
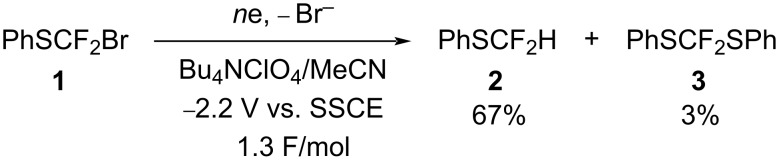
Cathodic reduction of **1**.

Next, we carried out the constant potential cathodic reduction of **1** at a platinum cathode in Bu_4_NClO_4_/MeCN. Notably, when 1.3 F/mol were passed, starting compound **1** was consumed completely. As shown in Scheme 4, difluoromethyl phenyl sulfide (**2**) was mainly formed as well as bis(phenylthio)difluoromethane (**3**) as a minor product. From these results, one-electron and two-electron reductions of **1** seem to take place simultaneously to generate radical and anionic intermediates.

In order to trap the radical intermediate, the constant potential cathodic reduction of **1** was performed in the presence of various olefins such as α-methylstyrene, cyclohexene, and dihydrofuran. The results are summarized in Table 1.

**Table 1:** Cathodic reduction of bromodifluoromethyl phenyl sulfide (**1**) in the presence of various olefins as a trapping reagent.

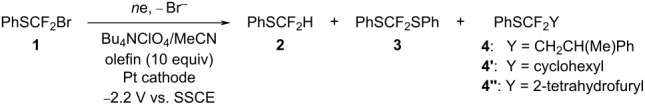							

Run	Olefin	Charge passed (F/mol)		Yield (%)^a^			

			**2**		**3**	**4–4’’**	

1	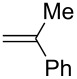	1.4	65 (60)^b^		trace	**4**	29 (23)^b^
2^c^	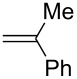	1.4	64		trace	**4**	12
3	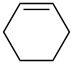	1.3	55		trace	**4’**	0
4	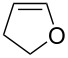	1.4	65		trace	**4’’**	0

^a^Determined by ^19^F NMR; ^b^isolated yield is shown in parentheses; ^c^glassy carbon cathode was used.

Regardless of trapping reagents, 1.3–1.4 F/mol of electricity was required to consume the starting material **1**. The required electricity was similar to the electrolysis in the absence of the trapping reagent. Only when α-methylstyrene was used as the radical trapping reagent, the expected radical adduct **4** was formed in reasonable yield of ca. 30% (Table 1, run 1). A platinum cathode is more suitable for the formation of adduct **4** compared to a glassy carbon cathode (Table 1, run 2). Dolbier et al. reported that electron-poor perfluoroalkyl radicals such as *n*-perfluoropropyl radical have high reactivity to electron-rich olefins such as α-methylstyrene and styrene [26]. In fact, our cathodically generated reactive species also reacted with α-methylstyrene. However, electron-rich dihydrofuran did not provide any radical adduct at all (Table 1, run 4). The reason is not clear at present. Thus the obtained results indicate that the cathodically generated reactive species would be the (phenylthio)difluoromethyl radical. In order to increase the yield of adduct **4**, the cathodic reduction of **1** was performed in other solvents such as DMF and CH_2_Cl_2_ using 20 equiv of α-methylstyrene. However, the yield of **4** did not increase.

The cathodic reduction of perfluoroalkyl halide generates radical and/or anionic species in general [24]. In order to generate radical species selectively, indirect cathodic reduction using various mediators has been often employed. Médebielle et al. successfully carried out the cathodic reduction of ArCF_2_X and RCOCF_2_X with nitrobenzene as a mediator to generate the corresponding difluoromethyl radicals selectively, and they applied this electrocatalytic system to the synthesis of various heterocyclic compounds bearing a perfluoroalkyl or perfluoroacyl group [27–30]. Furthermore, they extended this methodology to tandem cyclization to provide fused difluoromethylene-containing heterocycles [31]. In consideration of these facts, we studied the cathodic reduction of **1** using a mediator.

### Indirect cathodic reduction of **1** using *o*-phthalonitrile as mediator

**Figure 1:**
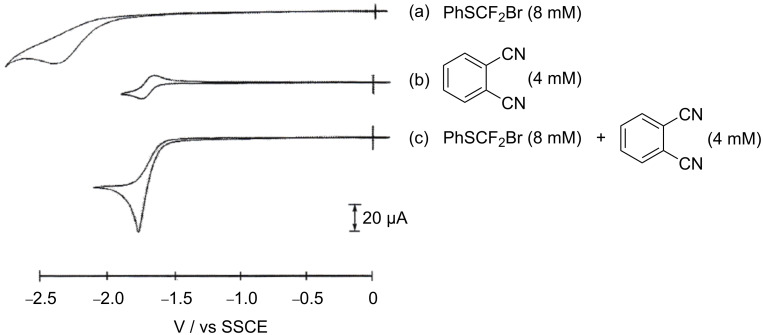
Cyclic voltammograms of (a) PhSCF_2_Br (**1**, 8 mM) in 0.1 M *n*-Bu_4_NClO_4_/MeCN; (b) *o*-phthalonitrile (4 mM), and (c) *o*-phthalonitrile (4 mM) + **1** (8 mM). Scan rate: 100 mV/s.

At first, cyclic voltammetry was carried out to investigate the electrocatalytic reduction of bromodifluoromethyl phenyl sulfide (**1**) with *o*-phthalonitrile as a mediator. The cyclic voltammograms of *o*-phthalonitrile in the absence and presence of compound **1** are shown in Figure 1.

As shown in Figure 1b, a typical reversible redox couple (*E*_1/2_^red^ = −1.69 V vs. SSCE) of *o*-phthalonitrile was clearly observed. A significantly enhanced cathodic peak current was observed after addition of compound **1** to the solution containing *o*-phthalonitrile while the anodic peak current disappeared completely as shown in Figure 1c. The reduction peak potential of **1** is −2.4 V vs. SSCE, which excludes the reduction of **1** at this potential. Therefore, the enhanced cathodic current of *o*-phthalonitrile clearly suggests that a typical electrocatalytic reduction reaction takes place. Thus, it was found that *o*-phthalonitrile should work as an electron transfer catalyst, i.e., a redox mediator.

**Scheme 5:**
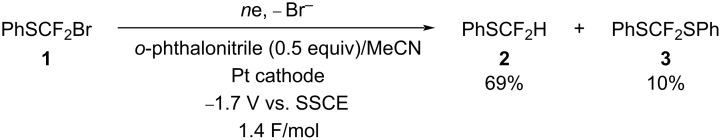
Indirect cathodic reduction of **1** using *o*-phthalonitrile as mediator.

On the bases of the cyclic voltammetric measurements, the cathodic reduction of **1** was carried out at a constant potential using *o*-phthalonitrile as mediator. As shown in Scheme 5, the total yield of products **2** and **3** increased appreciably to ca. 80% compared to the direct cathodic reduction of **1** (70% yield in Scheme 4).

Next, the indirect cathodic reduction of compound **1** was carried out similarly in the presence of α-methylstyrene and the results are summarized in Table 2.

**Table 2:** Indirect cathodic reduction of compound **1** using *o*-phthalonitrile as mediator in the presence of α-methylstyrene.

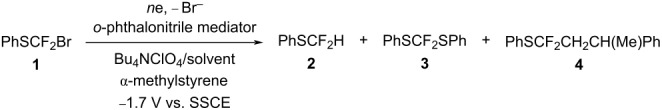						

Run	Solvent	Mediator	Charge passed (F/mol)	Yield (%)^a^		

				**2**	**3**	**4**

1	MeCN	–	1.4	65	trace	29
2	MeCN	0.2 equiv	1.5	44	trace	15
3	MeCN	0.5 equiv	1.8	26	trace	35
4	DMF	–	1.6	67	trace	16
5	DMF	0.5 equiv	1.9	31	trace	28

^a^Determined by ^19^F NMR.

When 0.2 equiv of the mediator were used, the yields of both products **2** and **4** were decreased compared to the direct cathodic reduction (Table 2, run 1). Increasing the amount of the mediator to 0.5 equiv resulted in an increase of the yield of **4** to 35% (Table 2, run 3) while the yield of **2** was decreased significantly. In this case, the required electricity was increased to 1.8 F/mol.

**Scheme 6:**
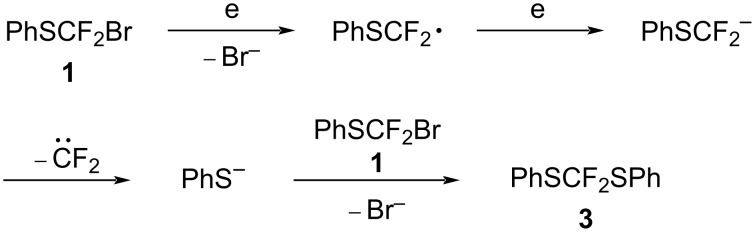
Mechanism for the formation of product **3**.

From these results, we anticipate that a one-electron reduction of compound **1** takes place to generate the PhSCF_2_ radical, which is further reduced affording the PhSCF_2_ anion when a trapping reagent is absent. The resulting anion seems to undergo elimination of difluorocarbene to generate a phenylthiolate anion which reacts with compound **1** to form product **3** as shown in Scheme 6.

**Scheme 7:**
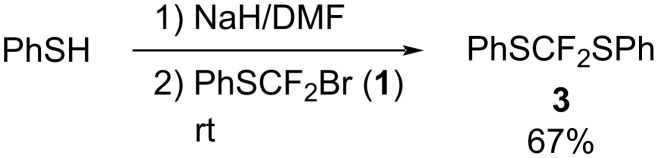
Reaction of compound **1** with PhS anions.

In order to confirm the proposed reaction pathway to product **3**, the reaction of bromodifluoromethyl phenyl sulfide (**1**) with phenylthiolate anion was performed at room temperature. As expected, product **3** was formed in moderate yield of 67% as shown in Scheme 7.

**Scheme 8:**
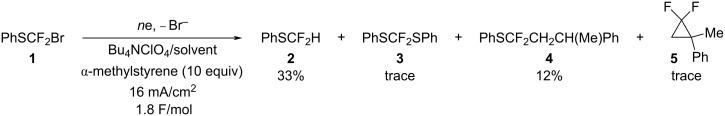
Cathodic reduction of compound **1** in the presence of α-methylstyrene at a high current density.

It is known that difluorocarbene has generally low reactivity towards olefins; however, it can be trapped with electron-rich olefins [32]. In order to trap difluorocarbene with an olefin, we tried to increase the amount of generated difluorocarbene by increasing the current density for the cathodic reduction of compound **1**. Thus, the cathodic reduction of **1** was carried out completely at a high current density of 16 mA/cm^2^ in the presence of α-methylstyrene. As shown in Scheme 8, the expected difluorocarbene adduct **5** was detected by high resolution mass spectrometry in addition to products **2**, **3**, and **4**.

As already mentioned, SmI_2_ is a well-known one-electron reducing reagent, and has been used to generate PhSCF_2_ radicals and perfluoroalkyl radicals from PhSCF_2_Br and perfluoroalkyl halides, respectively. The generated radicals undergo addition to olefins and acetylenes [5,33]. Pohmakotr et al. and Yoshida et al. reported that the reaction of PhSCF_2_Br and PhCF_2_Cl with SmI_2_ generated PhSCF_2_ and PhCF_2_ radicals, which were trapped with styrene [5,34–35]. Therefore, we carried out the reaction of compound **1** with SmI_2_ in the absence and presence of α-methylstyrene, which is more electron-rich compared to styrene. The results are summarized in Table 3.

**Table 3:** Reaction of compound **1** with SmI_2_ in the presence and absence of α-methylstyrene.

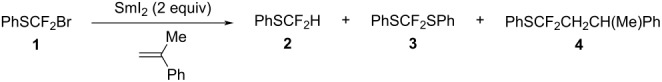						

Run	Solvent	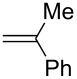 (equiv)	Conversion (%)	Yield (%)^a^		

				**2**	**3**	**4**

1	THF	0	34	12	trace	–
2	HMPA (7.5 equiv)/THF	0	66	5	32	–
3	HMPA (7.5 equiv)/THF	10	76	5	32	0
4	MeOH (10 equiv)/THF	10	45	5	0	14

^a^Determined by ^19^F NMR.

As shown in Table 3, even when two equivalents of SmI_2_ were used in the absence of α-methylstyrene, the conversion of compound **1** was low and a large amount of starting material **1** was recovered (Table 3, run 1). In this case, simple reduction product **2** was formed together with trace amounts of product **3**. Since HMPA is known to enhance the reducing ability of SmI_2_ [36], we performed the reaction of compound **1** in THF containing 7.5 equiv HMPA. As expected, the conversion of **1** increased from 34% to 66%, and product **3** was formed in 32% yield (Table 3, run 2). However, the yield of product **2** decreased from 12% to 5%. Then, the reaction of **1** with SmI_2_ was carried out similarly in the presence of α-methylstyrene (Table 3, run 3). However, the result was almost the same as that in the absence of α-methylstyrene: the yields of products **2** and **3** remained unchanged and the expected adduct **4** was not formed at all although the conversion of compound **1** increased. In both cases (Table 3, runs 2 and 3), unidentified products were formed. Thus, it was found that the chemical reduction of compound **1** with SmI_2_ was quite different from the electrochemical reduction. However, notably, when THF containing MeOH (10 equiv with regard to compound **1**) was used, adduct **4** was formed in 14% yield (Table 3, run 4). In this case, product **3** was not formed.

**Scheme 9:**
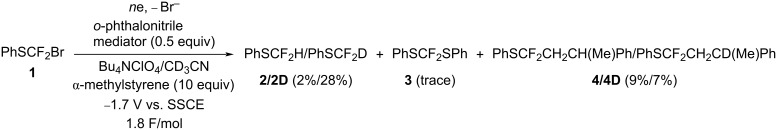
Indirect cathodic reduction of compound **1** in CD_3_CN.

In order to determine the hydrogen source of the products **2** and **4**, indirect cathodic reduction of **1** was carried out in deuterated acetonitrile, CD_3_CN (Scheme 9).

As shown in Scheme 9, deuterated products **2** and **4** were formed. In the case of product **2**, almost complete deuteration was observed, which clearly indicates that product **2** should be formed via a PhSCF_2_ radical intermediate. Thus, the main hydrogen source for the formation of product **2** was determined to be MeCN. On the other hand, in the case of adduct **4**, deuterated and protonated **4** were formed in a similar yield, which suggests that **4** would be formed via both radical and anionic intermediates.

In order to further clarify the reaction mechanism, the indirect cathodic reduction of compound **1** was performed in the presence of α-methylstyrene in MeCN containing cumene (iPrC_6_H_5_) and isopropyl alcohol (iPrOH). The former works as a hydrogen radical source while the latter works as both a hydrogen radical and proton source. The results are summarized in Table 4.

**Table 4:** Indirect cathodic reduction of compound **1** with *o*-phthalonitrile in the presence of α-methylstyrene in MeCN containing cumene or isopropyl alcohol.

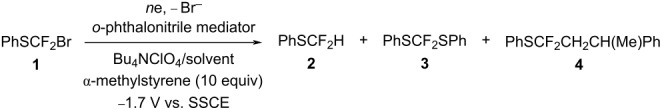					

Run	Solvent (hydrogen source)	Charge passed (F/mol)	Yield (%)^a^		

			**2**	**3**	**4**

1	MeCN	1.8	26	trace	35
2	MeCN/iPrC_6_H_5_ (10 equiv)	1.7	27	trace	23
3	MeCN/iPrOH (10:1)	1.7	15	0	44
4	MeCN/iPrOH (1:1)	2.7	20	0	60 (53)^b^

^a^Determined by ^19^F NMR; ^b^isolated yield is shown in parentheses.

**Scheme 10:**
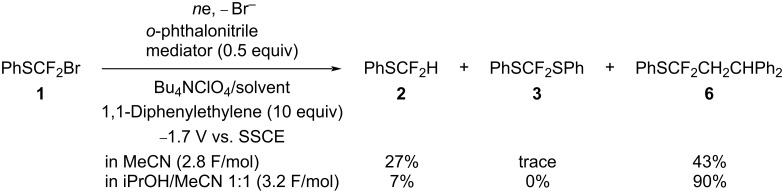
Indirect cathodic reduction of compound **1** in the presence of 1,1-diphenylethylene.

Although it was expected that the yield of product **4** would be increased in the presence of cumene as a hydrogen radical source, the yield was decreased (Table 4, run 2) compared to the electrolysis in the absence of cumene (Table 4, run 1). On the other hand, the yield of product **4** increased in the presence of iPrOH (Table 4, run 3), and the yield further increased to 60% at a higher content of iPrOH of 50% (Table 4, run 4). In the latter case, the required electricity was increased to 2.7 F/mol. Reutrakul and Pomakotr et al. also reported that iPrOH is an effective additive for the addition of PhSCF_2_ radical to olefins [5]. Since the presence of a large amount of a proton source such as iPrOH increased the yield of adduct **4** significantly, the electrolysis of compound **1** in the presence of 1,1-diphenylethylene as a more electron-rich olefin compared to α-methylstyrene was carried out similarly. As expected, the adduct **6** was formed in a high yield of 90% as shown in Scheme 10.

Isopropanol can serve as both a proton and a hydrogen radical source while cumene serves only as a hydrogen radical source. The indirect cathodic reduction of compound **1** in the presence of cumene decreased the yield of adduct **4** while the use of iPrOH instead of cumene increased the yield markedly. As already mentioned, in the chemical reduction of compound **1** with SmI_2_, only 10 equiv of MeOH to **1** also enhanced the formation of adduct **4** to some extent (from 0% to 14% yield) as shown in Table 3. Therefore, iPrOH seems to promote the radcal addition rather than reduction although the reason has not been clarified yet.

### Reaction mechanism

Although the cathodic reduction of perfluoroalkyl halides usually involves one- and two-electron transfer, their indirect cathodic reduction using mediators undergoes one-electron reduction selectively as reported by Saveant et al. [24]. In this study, we also confirmed that the *o*-phthalonitrile-mediated reduction of PhSCF_2_Br (**1**) in the absence of radical trapping reagents consumed much less than 2 F/mol of electricity. Furthermore, the indirect cathodic reduction of compound **1** in CD_3_CN formed the deuterated product, PhSCF_2_D (**2D**) as a major product. On the other hand, the indirect cathodic reduction of compound **1** in CD_3_CN containing a radical trapping reagent such as α-methylstyrene consumed less than 2 F/mol of electricity to provide protonated and deuterated adducts **4**/**4D** in almost same yields. Similar indirect electrolysis of compound **1** in iPrOH/MeCN in the presence of 1,1-diphenylethylene consumed much more than 2 F/mol of electricity to afford adduct **6** in high yield.

Moreover, the indirect cathodic reduction of compound **1** at high current density in the presence of α-methylstyrene formed a trace amount of 1,1-difluorocycopropane derivative **5**, which is an evidence of the generation of difluorocarbene from **1**.

**Scheme 11:**
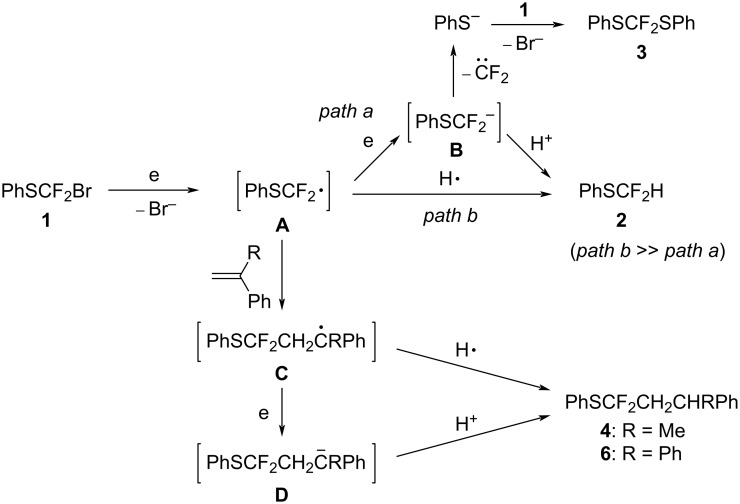
Reaction mechanism.

In consideration to these facts, we propose the following reaction mechanism as shown in Scheme 11.

The one-electron reduction of **1** generates the PhSCF_2_ radical **A**, which abstracts a hydrogen radical from MeCN to give product **2** (path b). The radical **A** undergoes further reduction to generate anion **B** (path a). Elimination of difluorocarbene from anion **B** forms a phenylthiolate anion, which reacts with the starting material **1** to form product **3**. In the presence of radical trapping reagents such as styrene derivatives, radical **A** reacts with styrenes to form radical intermediate adduct **C**. The radical **C** abstracts a hydrogen radical to form products **4** and **6**. Alternatively, the radical intermediate **C** is further reduced to generate anion **D** followed by protonation to give products **4** and **6**.

## Conclusion

We have successfully carried out catalytic electrochemical reduction of bromodifluoromethyl phenyl sulfide using *o*-phthalonitrile as mediator to generate (phenythio)difluoromethyl radicals selectively. The generated radicals were efficiently trapped with electron-rich olefins such as α-methylstyrene and 1,1-diphenylstyrene. The reaction mechanism was also disclosed by using the deuterated solvent CD_3_CN.

## Supporting Information

File 1 Experimental section: general information, materials, and general procedure for cathodic reduction of compound **1**.
