# Supplementary material for: Cathodic generation of reactive (phenylthio)difluoromethyl species and its reactions: mechanistic aspects and synthetic applications
Source: Beilstein J Org Chem. 2022 Jul 20;18:872–80. doi: 10.3762/bjoc.18.88 (PMC9344543; doi:10.3762/bjoc.18.88)
Supplement: File 1 — Experimental section: general information, materials, and general procedure for cathodic reduction of compound 1. [file Beilstein_J_Org_Chem-18-872-s001.pdf]

BEILSTEIN JOURNAL OF ORGANIC CHEMISTRY

Supporting Information

for

Cathodic generation of reactive (phenylthio)difluoromethyl species and its reactions: mechanistic aspects and synthetic applications

Sadanobu Iwase, Shinsuke Inagi and Toshio Fuchigami

Beilstein J. Org. Chem. **2022,** *18,* 872–880. doi:10.3762/bjoc.18.88

Experimental section: general information, materials, and general procedure for cathodic reduction of compound 1

License and Terms: This is a supporting information file under the terms of the Creative Commons Attribution License (https://creativecommons.org/licenses/by/4.0). Please note that the reuse, redistribution and reproduction in particular requires that the author(s) and source are credited and that individual graphics may be subject to special legal provisions.
The license is subject to the *Beilstein Journal of Organic Chemistry* terms and conditions: (https://www.beilstein-journals.org/bjoc/terms)

Experimental

General information

^{1}H, ^{13}C, and ^{19}F NMR spectra were recorded on a JEOL JNM EX-270 (^{1}H: 270 MHz, ^{13}C: 67.8 MHz, ^{19}F: 254.05 MHz) spectrometer in $CDCl_3$. The chemical shifts for ^{1}H NMR and ^{13}C NMR are given in δ (ppm) downfield from internal TMS and $CDCl_3$, respectively. ^{19}F NMR chemical shifts are given in δ (ppm) upfield from external trifluoroacetic acid. Oxidation potentials (E_p^{ox}) were measured in 0.1 M Bu_4NClO_4/MeCN at a scan rate of 100 mV/s by cyclic voltammetry using an ALS Instruments model 600 A. A platinum electrode (φ = 1 mm) and a platinum wire (φ = 1 mm × 30 mm) were used as working and counter electrodes, respectively. A saturated sodium chloride calomel electrode (SSCE) was used as a reference electrode. Preparative electrolysis experiments were carried out with a Hokuto Denko HABF 501 potentiostat/galvanostat. Mass spectra and high resolution mass spectra were obtained with a Shimadzu GCMS-QP-2000A or JEOL JMS-700 mass spectrometer. Deuterium incorporation to products **2** and **4** were determined by MS analyses. ^{19}F NMR yields were estimated with α,α,α-trifluorotoluene as an internal standard.

Materials

Dry MeCN was obtained by distillation in the presence of CaH_2 under nitrogen gas. THF and ether were dried over Na/benzophenone under reflux and distilled under nitrogen gas. The starting material, bromodifluoromethyl phenyl sulfide (**1**) was prepared by the modification of the known procedures [1] as follows.

Thiophenol (0.1 mol) was added dropwise to a stirred solution of 100 mL of dry THF containing NaH (0.11 mol) under a nitrogen atmosphere at room temperature. After 30 min, the solution was cooled to −10 °C, and cooled CBr_2F_2 (0.11 mol) was added dropwise to the solution of PhSNa in THF using a syringe. After 2 h, the solution was warmed to room temperature and stirred for 1 h. The solution was mixed with water, the product was extracted with ether for three times, and then the combined extracts were washed with brine followed by drying with anhydrous Na_2SO_4. The solvent was removed by evaporation, the remaining oil was distilled at 54 °C under reduced pressure (3 mmHg) to give bromodifluoromethyl sulfide (**1**) in 85% yield.

^{1}H NMR, δ = 7.38-7.68 (m, 5H, C_6H_5); ^{19}F NMR δ = 54.43 (s, 2F, SCF_2Br); ^{13}C NMR δ = 119.25, 127.17, 129.43, 131.03, 136.37; MS, 240 (M^++2), 238 (M^+), 159 (M^+-Br), 109 (M^+-CF_2Br), 77 (Ph^+).

General procedure for the cathodic reduction of 1

Constant current and constant potential electrochemical reduction of **1** (2.5 mmol) was carried out with a platinum cathode and anode (2 cm × 2 cm) in a divided glass cell with a glass frit separator containing 25 mL of 0.8 M Bu_4NClO_4/MeCN in the presence or absence of trapping olefins (10 mmol) such as α-methylstyrene and 1,1-diphenylethylene as a catholyte until **1** was completely consumed (monitored by TLC and GC–MS). After electrolysis, the NMR yield of products **2**–**5** were estimated from ^{19}F NMR spectra using $CF_3C_6H_5$ as an internal standard. After the estimation, the crude mixture was passed through a short column with silica gel to remove the supporting electrolyte, and the eluent was subjected to PTLC (eluent: hexane/AcOEt 9:1), and products **2**–**4** and **6** were isolated. 1,1-Difluoro-2-methyl-2-phenylcycopropane (**5**) [2]

was detected by MS and HRMS spectra. MS: m/z 168 (M^+), 149 (M^+−F), 77 (Ph^+); HRMS: m/z calcd for $C_{10}H_{10}F_2$: 168.0751, found 168.0743.

The known compounds, **2** [3] and **3** [4] were identified by comparison of the spectral data with those reported in the literature.

1,1-Difluoro-1-phenylthio-3-phenylbutane (**4**).

^{1}H NMR, δ = 7.19–7.58 (m, 10H, C_6H_5), 3.21 (m, 1H, C*H*Ph), 2.43 (m, 2H, CF_2CH_2), 1.35 (d, *J* = 7.3 Hz, 3H, CH_3); ^{19}F NMR δ = 7.2 (dd, *J* = 204, 16 Hz, 1F, SCF*F* CH_2), 4.8 (ddd, *J* = 206, 15, 12 Hz, 1F, C*F*FCH_2); MS: m/z 278 (M^+), 159 ($PhSCF_2^+$), 153 (M^+−$PhCHCH_3$), 109 (PhS^+), 105 (M^+−$PhSCF_2$ CH_2), 77 (Ph^+); HRMS: m/z calcd for $C_{16}H_{16}F_2S$: 278.0941, found 278.0940.

1,1-Difluoro-1-phenylthio-3,3-diphenylpropane (**6**)

^{1}H NMR, δ = 7.18–7.54 (m, 15H, C_6H_5), 4.41 (t, *J* = 6.9 Hz,1H, Ph_2CH CH_2), 2.96 (dt, *J* = 15.0 Hz, 2H, CF_2CH_2CH); ^{19}F NMR δ = 5.96 (t, *J* = 14.7, 2F, SCF_2 CH_2); MS: m/z 340 (M^+), 230 (M^+−PhSH), 152 (M^+−PhSH-PhH), 109 (PhS^+), 77 (Ph^+); HRMS: m/z calcd for $C_{21}H_{18}F_2S$: 340.1097, found 340.1094.

Deuterated products **7** and **8** were identified by ^{19}F NMR and MS spectra as follows.

α-Deuterio-α,α-difluoromethyl phenyl sulfide (**2D**)

^{19}F NMR δ = −15.83 (t, *J* = 8.7 Hz, 1F, SCF_2D); MS: m/z 161 (M^+), 111 (M^+−CF_2), 109 (PhS^+), 77(Ph^+).

3-Deuterio-1,1-difluoro-1-phenylthio -3-phenylbutane (**4D**)

^{19}F NMR δ = 7.1 (m, 1F, SCF*F* CH_2), 4.8 (dt, *J* = 203, 14 Hz, 1F, SC*F*F CH_2); MS: m/z 279 (M^+), 259 (M^+−HF), 109 (PhS^+), 106 (M^+−$PhSCF_2CH_2$), 77 (Ph^+).

References

1. Toulgoat, F.; Langlois, B. R.; Médebielle, M.; Sanchez, J.-Y. *J. Org. Chem*. **2007**, *72*, 9046-9052. doi: 10.1021/jo701318n
2. Dolbier W. R., Jr.; Wojtowicz, H.; Burkholder, C. R. *J. Org. Chem*., **1990**, *5*5, 5420–5422. doi: 10.1021/jo00306a027
3. Burton, D. J.; Wiemers, D. M. *J. Fluorine Chem*., **1981**, *18*, 573–582. doi: 10.1016/S0022-1139(00)82673-1
4. Rico, I.; Wakselman, C. *Tetrahedron Lett.* **1981**, *22*, 323–326. doi: 10.1016/0040-4039(81)80087-1
